# Free concepts association: a neural model

**DOI:** 10.1186/1471-2202-12-S1-P339

**Published:** 2011-07-18

**Authors:** Eleonora Russo, Alessandro Treves

**Affiliations:** 1 Cognitive Neusoscience Sector, SISSA, Trieste, 34136, Italy

## 

The investigation of complex cognitive functions can not leave aside the study of general collective behaviours generated by the presence of interacting units.

In this study we focus on the dynamics underlying a free association between two memories. At the computational level this process can be viewed as a spontaneous jump between attractive configurations of a Potts network, an autoassociative network sketching out the salient features of interacting patches of cortex. In the net each constitutive unit stands for a local patch of cortex. Thus, the connection of these units can be regarded as a global network of local subnetworks.

In the system the jump between two memories is elicited by an adaptive process, which weakens the stability of memories representation. The “latching process” so generated results a spontaneous consecutive retrieval of stored memories.

Acting on the parameters of the model (such the units connectivity, the number of stored memories, the noise) it is possible to explore a variety of behaviours that goes from a “finite latching” region, in which the system, governed by its relaxation time and the global memories structure, allows only finite sequences of memories retrievals, to an “infinite latching” region, in which this jumping dynamics go on indefinitely.

